# ZnO Nanoparticles of *Rubia cordifolia* Extract Formulation Developed and Optimized with QbD Application, Considering Ex Vivo Skin Permeation, Antimicrobial and Antioxidant Properties

**DOI:** 10.3390/molecules27041450

**Published:** 2022-02-21

**Authors:** Jasmeet Kaur, Md. Khalid Anwer, Ali Sartaj, Bibhu Prasad Panda, Abuzer Ali, Ameeduzzafar Zafar, Vinay Kumar, Sadaf Jamal Gilani, Chandra Kala, Mohamad Taleuzzaman

**Affiliations:** 1 Department of Pharmacognosy and Phytochemistry, School of Pharmaceutical Education and Research, Jamia Hamdard, New Delhi 110062, India; jasmeetkaur_sch@jamiahamdard.ac.in; 2 Department of Pharmaceutics, College of Pharmacy, Prince Sattam bin Abdulaziz University, P.O. Box 173, Al-Kharj 11942, Saudi Arabia; m.anwer@psau.edu.sa; 3 Department of Pharmaceutics, Faculty of Pharmacy, School of Pharmaceutical Education and Research, Jamia Hamdard, New Delhi 110062, India; sartaz005@gmail.com; 4 Microbial and Pharmaceutical Biotechnology Laboratory, Department of Pharmacognosy and Phytochemistry, School of Pharmaceutical Education and Research, Jamia Hamdard, New Delhi 110062, India; bppanda@jamiahamdard.ac.in; 5 Department of Pharmacognosy, College of Pharmacy, Taif University, P.O. Box 11099, Taif-21944, Saudi Arabia; abuali@tu.edu.sa; 6 Department of Pharmaceutics, College of Pharmacy, Jouf University, Al-Jouf 72341, Saudi Arabia; zzafarpharmacian@gmail.com or; 7 Department of Pharmacology, KIET School of Pharmacy, Delhi-NCR, Meerut Road (NH-58), Ghaziabad 201206, India; vinaykumarpatel@gmail.com; 8 Department of Basic Health Sciences, Preparatory Year, Princess Nourah Bint Abdulrahman University, Riyadh 11671, Saudi Arabia; SJGlani@pnu.edu.sa; 9 Department of Pharmacology, Faculty of Pharmacy, Maulana Azad University, Village Bujhawar, Tehsil Luni, Jodhpur 342802, India; ckpharmacology@gmail.com; 10 Department of Pharmaceutical Chemistry, Faculty of Pharmacy, Maulana Azad University, Village Bujhawar, Tehsil Luni, Jodhpur 342802, India

**Keywords:** ZnO-MJE nanoparticles, Box–Behnken design, permeation study, antimicrobial and antioxidant activity, in vitro release

## Abstract

The objective of the current research is to develop ZnO-Manjistha extract (ZnO-MJE) nanoparticles (NPs) and to investigate their transdermal delivery as well as antimicrobial and antioxidant activity. The optimized formulation was further evaluated based on different parameters. The ZnO-MJE-NPs were prepared by mixing 10 mM ZnSO_4_·7H_2_O and 0.8% *w*/*v* NaOH in distilled water. To the above, a solution of 10 mL MJE (10 mg) in 50 mL of zinc sulfate was added. Box–Behnken design (Design-Expert software 12.0.1.0) was used for the optimization of ZnO-MJE-NP formulations. The ZnO-MJE-NPs were evaluated for their physicochemical characterization, in vitro release activity, ex vivo permeation across rat skin, antimicrobial activity using sterilized agar media, and antioxidant activity by the DPPH free radical method. The optimized ZnO-MJE-NP formulation (F13) showed a particle size of 257.1 ± 0.76 nm, PDI value of 0.289 ± 0.003, and entrapment efficiency of 79 ± 0.33%. Drug release kinetic models showed that the formulation followed the Korsmeyer–Peppas model with a drug release of 34.50 ± 2.56 at pH 7.4 in 24 h. In ex vivo studies ZnO-MJE-NPs-opt permeation was 63.26%. The antibacterial activity was found to be enhanced in ZnO-MJE-NPs-opt and antioxidant activity was found to be highest (93.14 ± 4.05%) at 100 µg/mL concentrations. The ZnO-MJE-NPs-opt formulation showed prolonged release of the MJE and intensified permeation. Moreover, the formulation was found to show significantly (*p* < 0.05) better antimicrobial and antioxidant activity as compared to conventional suspension formulations.

## 1. Introduction

**Figure 1:**
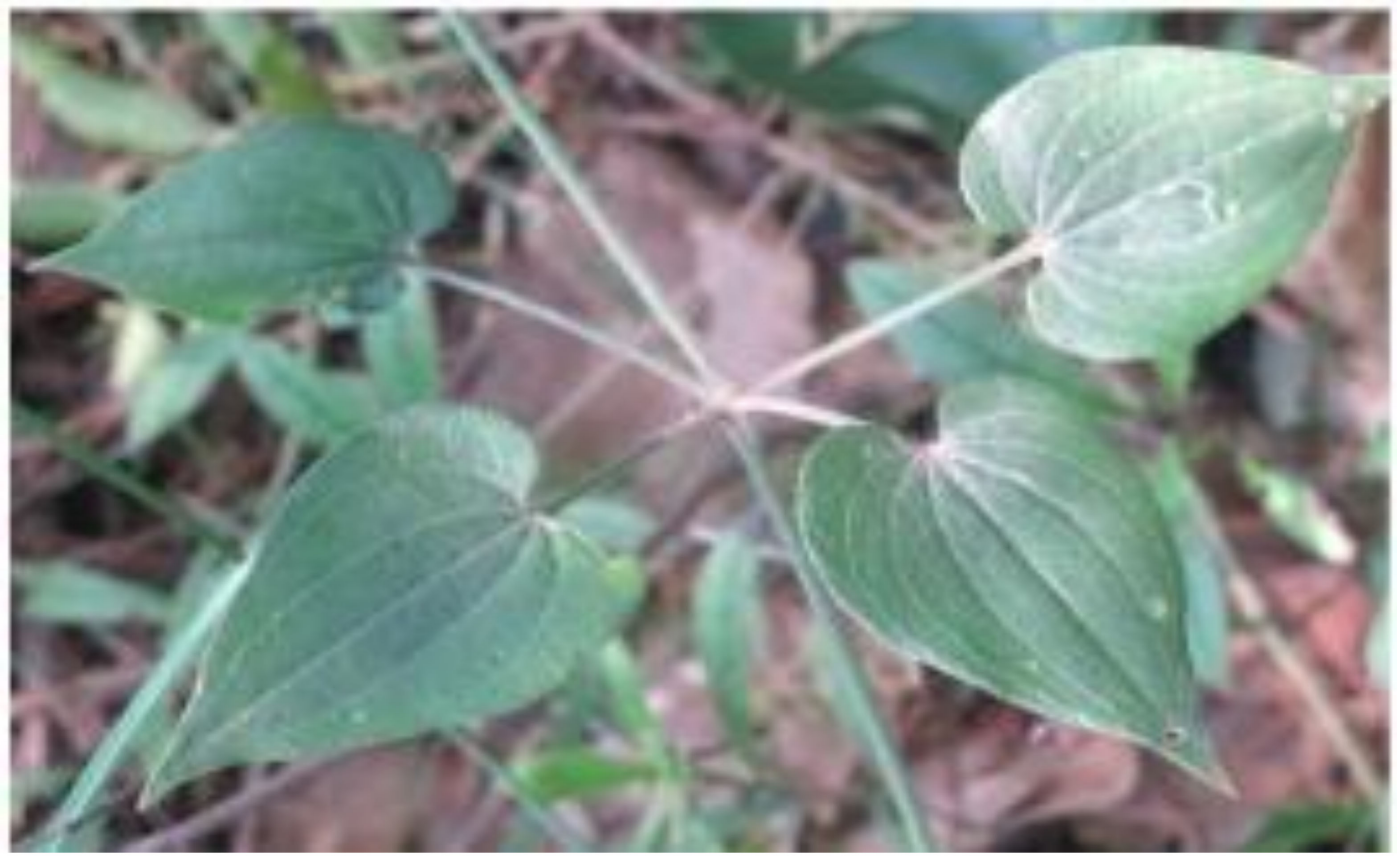
*Rubia cordifolia* (Image taken under Creative commons CC by 4.0 license).

*Rubia cordifolia* (Figure 1), a medicinal plant commonly known as Manjistha, has gained increased clinical attention because of its high therapeutic potential [1].

Globally, it has been used for the conventional treatment of skin problems/disorders, stimulation of lymphatic flow, and blood disorders [2]. The root of the plant has been used traditionally for the treatment of rheumatism, menstrual disorders, jaundice, urinary disorders, renal stones, skin disorders, and blood detoxification. Previous studies have reported its antioxidant activity and enhanced production of antioxidant markers such as superoxide dismutase, catalase, and glutathione [3,4], along with the anti-inflammatory activity of Manjistha extract (MJE) [5,6]. Besides its medicinal value, the plant is used in food, syrups, and medicated oils as a natural colorant. Moreover, the leaf extract is widely used for cataracts, eczema, psoriasis, and in the treatment of chikungunya fever. Herbal extracts containing flavonoids, tannins, and terpenoids are soluble in water but the drawback is low absorption, which affects the bioavailability and efficacy [6]. Nanotherapeutics is a desired drug-targeted goal to provide high safety and efficacy over the conventional system [7,8,9].

A nanocarrier delivery system has been adopted to overcome the disadvantages of repeated dosage administration [10]. Nanotechnology-based drug delivery systems including liposomes, ethosomes, niosomes, nano-emulsions, micro-emulsions, nanostructured lipid carriers, polymeric micelles, and inorganic nanoparticles have been reported to enhance the oral bioavailability of many medicines [11,12,13,14,15]. The nanoformulation size, charge, and surface morphology play important roles in the physicochemical properties and therapeutic potential of drugs [16]. A colloidal dispersion of nanoparticles formulates the nanosuspension and is stabilized by a surfactant [17]. Inorganic nanoparticles using ZnO have significant therapeutic potential. ZnO has various advantages like low cost, biocompatibility, nontoxicity, antimicrobial property, and high availability in nature [18]. The green synthesis of nanoparticles using *Rubia cordifolia* was reported earlier for zinc oxide nanoparticles (ZnO-NPs) and silver nanoparticles (Ag-NPs) with antibacterial activity [19,20]. Previous studies have reported that the ZnO nanoparticles can be used as an herbal bioactive compound to increase the therapeutic potential of plants like *Calotropis procera* [21], *Poncirus trifoliata* [22], and *Artemisia annua* [23].

The formulation development process uses optimization with the approach of quality by design (QbD) to understand the better variable effects and robust quality of the product to confirm the target quality product profile. Design of experiment (DoE) requires critical parameters with fixed ranges to obtain design space (DS) based on the risk assessment process and formulation variables [24,25,26,27].

In the surface response methodology, the Box–Behnken design (BBD) includes powerful, effective, and systematic tools that lessen the time required for the development of pharmaceutical dosage forms with enhanced research output. BBD permits the designer to utilize three levels of each factor to properly achieve the quantification.

This current study focuses on the development of the formulation of ZnO-NPs of Manjistha extract. Optimization of the formulation was performed by Design-Expert software considering variable parameters such as particle size, % entrapment efficiency, and polydispersity index (PDI), and the independent variables were the concentration of ZnSO_4_·7H_2_O (% *w*/*v*), stirring speed (rpm), and ultrasonic time (minutes). Thereafter, the optimized formulation was analyzed for morphology, particle size, zeta potential, in vitro drug release, ex vivo skin study, stability study, and antimicrobial as well as anti-oxidant activities. The objective of the association of ex vivo study with antimicrobial and anti-oxidant activity was to enhance the therapeutic effect by increasing the drug release to reach systemic circulation quickly.

## 2. Experimental Work

### 2.1. Materials

Manjistha extract was procured from the Sunpure Research Incubation Center, Sunpure Extracts Pvt. Ltd. Delhi, India. The chemicals zinc sulfate (ZnSO_4_·7H_2_O) and sodium hydroxide (NaOH) were of analytical grade and were procured from SD Fine Chemical (Mumbai, India).

### 2.2. Preparation of Nanoparticles

**Scheme 1:**
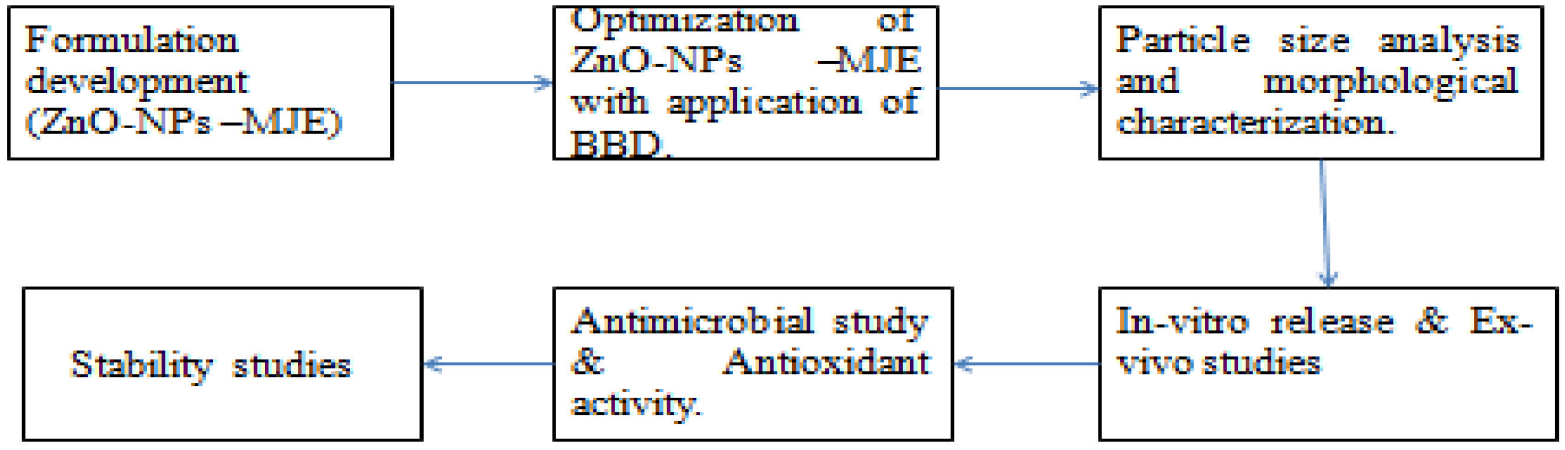
Flow chart of work.

The preparation of ZnO-NPs of an aqueous MJE was reported previously [28]. A solution of zinc sulfate (ZnSO_4_·7H_2_O) and sodium hydroxide (NaOH) was prepared with a strength of 10 mM (5.75% *w*/*v* and 0.8% *w*/*v* in distilled water, respectively) as shown in the Scheme 1. Ten milliliters of MJE (1 mg/mL) were transferred to 50 mL of zinc sulfate solution; then, NaOH was added dropwise until a white suspension of nanoparticles was produced. Nanoparticles were centrifuged at 5000 rpm for 10 min and then stored in the refrigerator.

### 2.3. Box–Behnken Design (BBD) Optimization

The ZnO-MJE-NPs were optimized using Box–Behnken design (Design-Expert software version 12.0.1.0). Depending upon the preliminary study, the concentration of ZnSO_4_·7H_2_O (% *w*/*v*), stirring speed (rpm), and ultrasonic time (minutes) were selected as the independent variables, and particle size, PDI, and entrapment efficiency were selected as dependent variables. The software found 13 formulations as expressed in Table 1. The data of each response were fitted into different experimental design models for obtaining the best-fit model. The 3-D plot of each response was constructed, which showed the graphical presentation of independent variables over the responses [29,30].

### 2.4. UV-Spectrophotometer Analysis

A standard stock solution of MJE was prepared by accurately dissolving 10 mg of MJE in 10 mL of phosphate buffer at pH 7.4. The calibration curve of MJE was plotted within the concentration range of 5–60 µg/mL, with a linearity equation of Y = 0.0147x − 0.0272, and its R^2^ value 0.9954 at 285 nm. The limit of detection (LOD) and limit of quantification (LOQ) values were 1.7 and 5 µg/mL, respectively [4,19].

### 2.5. Particle Size Analysis and Morphological Characterization

#### 2.5.1. Particle Size and Polydispersity Index (PDI)

The Zetasizer-1000 HS (Malvern Instruments, Malvern, UK) was used to study particle size and PDI. The particle size and PDI were measured with dynamic light scattering (DLS). Water was used to dilute the sample and then placed in a quartz cuvette and analyzed at a 90° scattering angle. All of the batches were analyzed in triplicate, then mean and standard deviation (SD) value was calculated [31,32].

#### 2.5.2. Zeta Potential

Zeta potential is an important parameter to predict the stability of the particulate formulation. The electric charge is reflected on the particle surface, which indicates the physical stability of the nanoparticle. It was measured by the Zetasizer-1000 HS (Malvern Instruments, Malvern, UK) [33].

#### 2.5.3. Structural Analysis by TEM

Transmission Electron Microscopy (TEM Tecnai, G20, Philips Scientific, Amsterdam The Netherlands) was employed to study the shape of the developed nanoparticle. The sample was taken in the carbon-coated grid and stained with phosphotungstic acid under the microscope at 10–100 k times enlargements at 200 kV voltage [34].

#### 2.5.4. Entrapment Efficiency

The ultracentrifugation method was employed to determine the entrapment efficiency of MJE in the ZnO-MJE-NPs. The ZnO-MJE-NPs were centrifuged at 18,000 rpm and 4 °C for 15 min. The supernatants were collected and free MJE concentration was analyzed at 285 nm by a UV-spectrophotometer (Shimadzu-1601, New Delhi, India). The below mathematical formula was used to calculate the % entrapment efficiency [35].

(1) $$\%\;\mathrm{Entrapment}\;\mathrm{efficiency}=\frac{\mathrm{Total}\;\mathrm{MJE}-\mathrm{MJE}\;\mathrm{in}\;\mathrm{supernatant}}{\mathrm{Total}\;\mathrm{MJE}}\times 100$$

### 2.6. In Vitro Release Studies

In vitro release of the ZnO-MJE-NPs and MJE dispersion was performed at pH 7.4 using a dialysis membrane (molecular weight cut-off 10–12 KDa). Three milliliters of ZnO-MJE-NPs and MJE dispersion (15 mg of MJE) was filled into a dialysis bag and placed in 100 mL dissolution media of different pH. The dissolution medium was stirred regularly at 100 rpm and the temperature was maintained at 37 °C on a magnetic stirrer. The drug content was measured by UV-spectrophotometer. A total of 2 mL of aliquots of the above solution was taken at a different time intervals (5, 10, 30 min; 1, 2, 4, 8, 12, and 24 h) and was replaced with the same amount of dissolution medium to maintain the constant volume and concentration gradient [36,37]. The % drug releases were calculated and plotted on the graph. The drug release data were fitted to different kinetic models such as zero order, first order, Higuchi, and Korsmeyer–Peppas to evaluate the release mechanisms from the ZnO-NPs [38].

### 2.7. Ex Vivo Studies

A Franz diffusion apparatus (9 mm orifice diameter and receptor volume 5 mL) was used to conduct the ex vivo permeation study using rat skin (IAEC/KSOP/E/20/03, Registration No 1099/PO/Re/S/CPCSEA). The stratum corneum with dermal layer in the downwards direction was placed in between the two compartments, i.e., donor and receptor. Tyrode solution pH 7.4 was used as the permeation medium and filled into the receptor compartments [16]. A total of 1 mL (2 mg drug) of ZnO-MJE-NPs and MJE dispersion was poured into the donor cell, and temperature was maintained at 37 ± 0.5 °C throughout the study. At specific time intervals, 1 mL of released content was taken and analyzed by UV-spectrophotometer [39] at 285 nm. The flux, permeability coefficient (PC), and enhancement ratio (ER) were calculated.

(2) $$\mathrm{Permeability}\;\mathrm{coefficient}=\frac{\mathrm{Flux}}{\mathrm{Area}\;\times \mathrm{total}\;\mathrm{amount}\;\mathrm{of}\;\mathrm{MJE}}$$

(3) $$\mathrm{ER}=\frac{\mathrm{PC}\;\mathrm{of}\;\mathrm{formualtion}}{\mathrm{PC}\;\mathrm{of}\;\mathrm{pure}\;\mathrm{drug}}$$

### 2.8. Antimicrobial Study

The antimicrobial activity of the prepared ZnO-MJE-NPs was evaluated against the tested organisms (*S. Aureus*, and *E. Coli*) using the pour plate method. The study was performed in the sterilized agar medium. The test samples were dispersed in sterilized water [40]. The prepared melted sterilized nutrient agar medium was transferred and mixed with the microorganism in Petri plates and allowed to stand for solidification without any agitation. The well (10 mm) was made using a sterilized borer, and 1 mL of varied concentrations of the drug (20–320 µg/mL of MJE) was added. The Petri plates were set aside for a few hours and then placed into an incubator for 24 h. The study was conducted in triplicate and ZOI was noted using a graduated scale in millimeters (mm).

### 2.9. Antioxidant Activity

The percentage of antioxidant activity (% AA) of ZnO-MJE-NPs and MJE dispersion was assessed by 2, 2-Diphenyl-1-picrylhydrazyl (DPPH) free radical scavenging method as described by Brand-Williams et al. [41]. The 0.02% DPPH solution was prepared in ethanol. ZnO-MJE-NPs and MJE dispersion at 10–100 µg/mL from the stock solution (1 mg/mL). A total of 500 µL of each concentration of ZnO-MJE-NPs and MJE dispersion was added into the DPPH solution (125 µL) and let stand for 1 h in a dark place. The change in color from violet to colorless indicated antioxidant activity. The absorbance (Abs) was read at 517 nm using a UV-VIS spectrophotometer (DU 800; Beckman Coulter, Fullerton, CA, USA). A DPPH solution without ZnO-MJE-NPs and MJE dispersion was taken as blank. The % AA was calculated by the below equation.

(4) $$\%\;\mathrm{AA}=\frac{\mathrm{Absorbence}\;\mathrm{of}\;\mathrm{control}\;\mathrm{sample}-\mathrm{Absorbance}\;\mathrm{of}\;\mathrm{test}\;\mathrm{sample}}{\mathrm{Absorbence}\;\mathrm{ofcontrol}\;\mathrm{sample}}\times 100$$

### 2.10. Stability Studies

A stability study was performed to assess the change in formulation during storage or shelf life. The optimized ZnO-MJE-NP formulation was sealed in glass vials and placed into a stability chamber at 25 ± 1 °C/60% RH for 3 months. Samples were taken at 0, 1, 2, and 3 months and were evaluated for their physical appearance, particle size, and entrapment efficiency [42].

### 2.11. Statistical Analysis

All the data were expressed in mean ± SD. The Graph Pad prism software was used for statistical analysis. Student’s *t* test was used to assess statistical significance at *p* ≤ 0.005 for all the parameters. A statistical significance at *p* < 0.001 was used to assess antimicrobial activity.

## 3. Results and Discussion

### 3.1. Optimization

#### 3.1.1. Effect of independent variables on particle size

The polynomial equation for particle size is given below.

Particle Size = +259.38 − 0.15A − 0.90B − 0.52C + 0.50AB + 2.45AC − 2.30BC + 2.62A² + 0.62 B² − 0.41C²(5)

**Figure 2:**
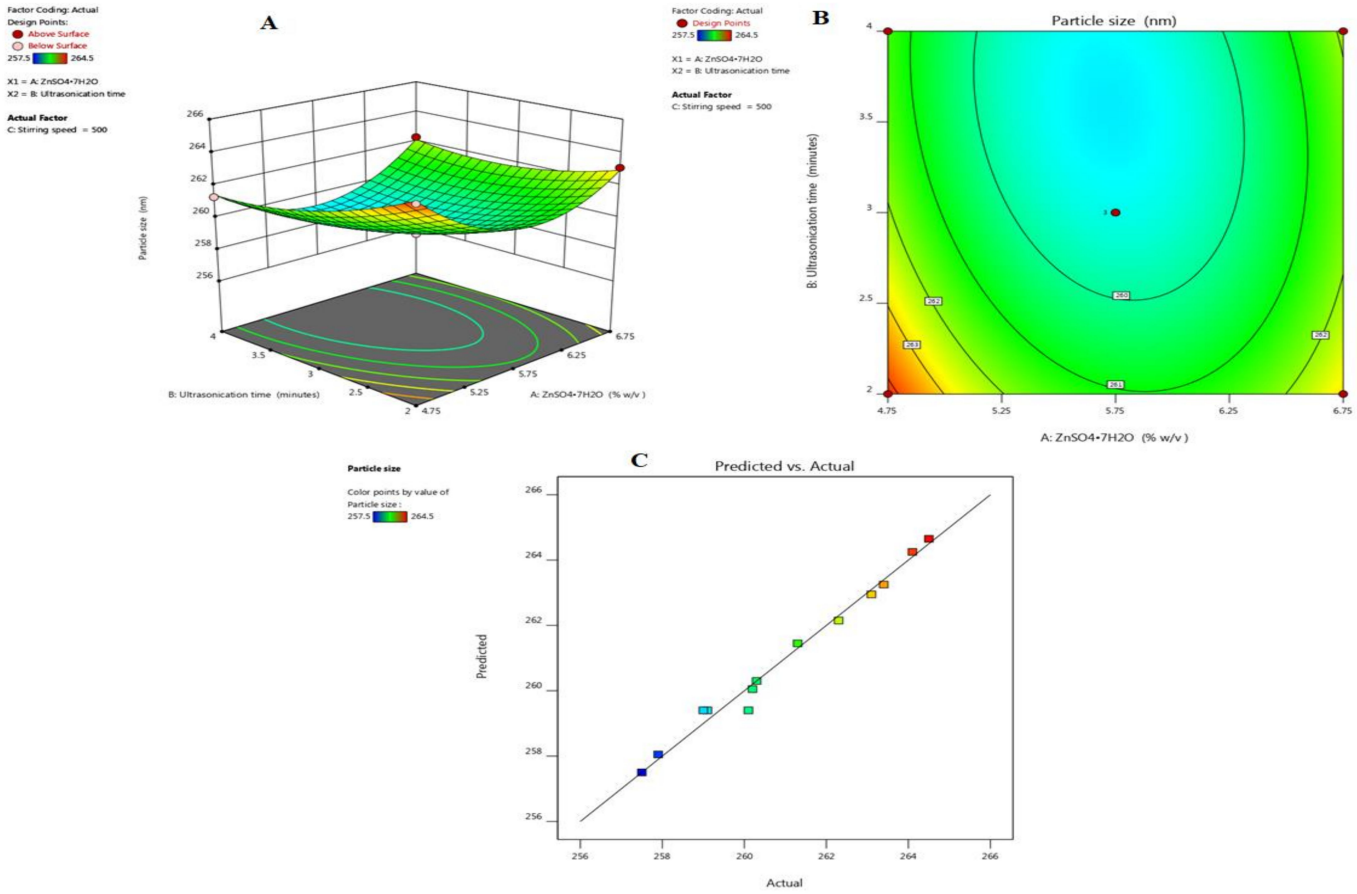
3D (**A**), contour (**B**), and actual and predicted (**C**) values of particle size.

The results of independent variables on the particle sizes of different formulations are given in Table 1. Negative and positive signs indicate the favoring and un-favoring of the independent variable over the response. This equation showed B, AC, BC, A² to be significant model terms because *p* < 0.05. The model F-value of 23.30 (*p* < 0.05) indicated that the model was significant. The quadratic model was evaluated based on an ANOVA and indicated that it was significantly fitted (Table 2). The F-value of lack of fit of 0.49 indicated that it was non-significant, which was good for the model. The R^2^ of the quadratic model was 0.9859, which was greater than the other models. The adequate precession greater than 4 (14.72) indicated an adequate signal. The ZnSO_4_ showed the positive effect on particle size. An increase in the concentration of ZnSO_4_ resulted in increased particle size because of an increase in the viscosity of the dispersion. The sonication time (B) exhibited a negative effect on particle size. The size of the nanoparticle decreased with increasing sonication time because of the breaking of particles. The stirring speed (C) exhibited a negative effect on the particle size, which meant that with increased stirring speed the particle size decreased from the breaking of the particle, but this was not affected significantly. Graphs of the 3D, contour, and actual and predicted values of the particle size are shown in Figure 2A–C.

#### 3.1.2. Effect of Independent Variables on Polydispersity Index (PDI)

**Figure 3:**
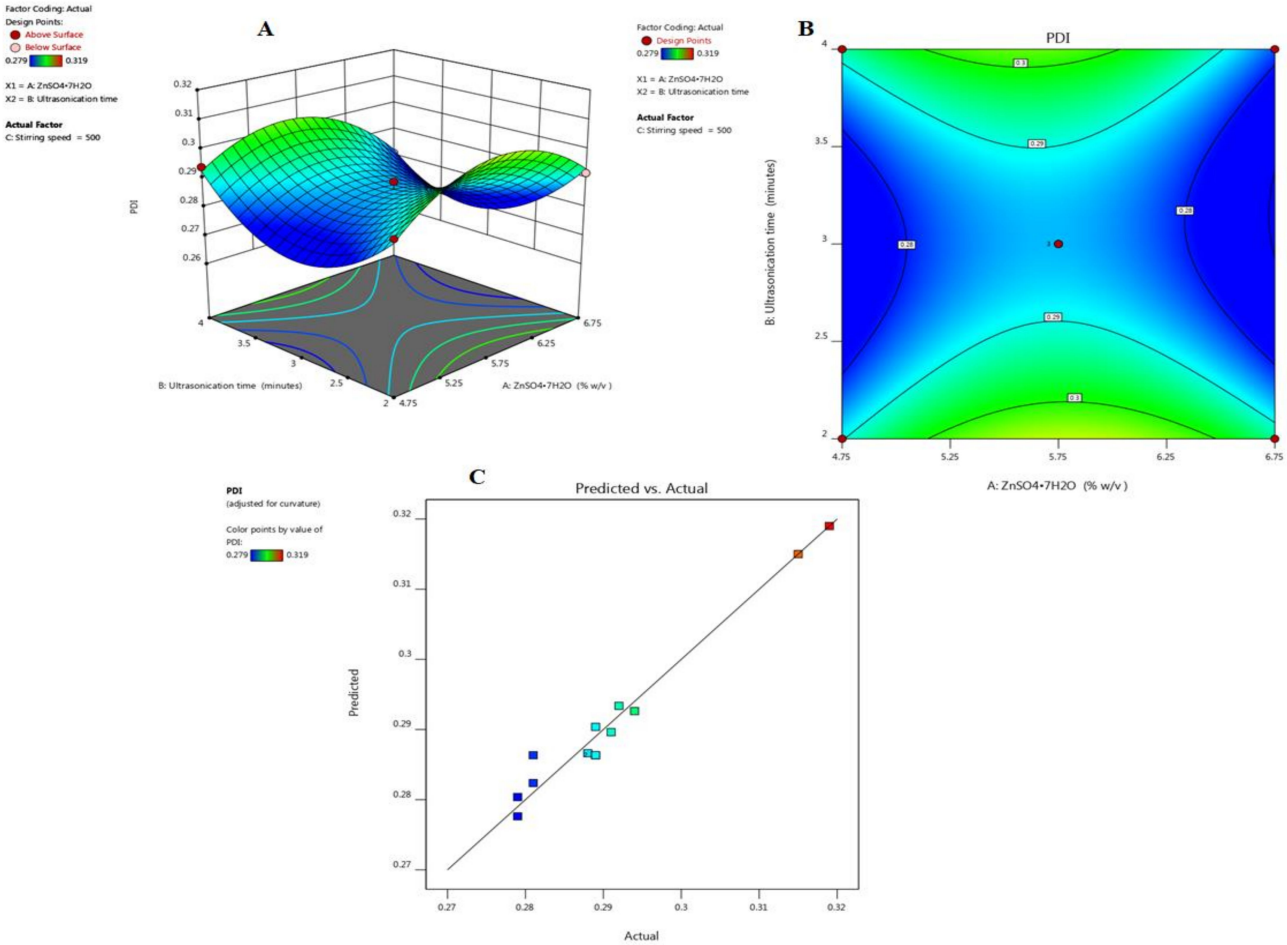
3D (**A**), contour (**B**), and actual and predicted (**C**) values of particle size.

The effects of independent variables on the PDI of different formulations is given in Table 1. The experimental design showed that the quadratic model was found to be the best fitted model. The F-value of 10.50 indicated that the model was significantly fitted. The lack of fit F-value of 0.71 implied the lack of fit was not significant relative to the pure error. There was 48.84% chance that a lack of fit F-value of 0.71 could occur because of noise. Non-significant lack of fit was good. The R^2^ of 0.9692 was greater than other models (linear, second order), indicating that model was well fitted. The adequate precession of 10.74 (>4) revealed that model was significantly fitted. An ANOVA of the quadratic model was calculated and is given in Table 2. Graphs of the 3D, contour, and actual and predicted values of particle size are displayed in Figure 3A,B, showing the effect of the independent variables on the PDI. The actual and predicted values of the PDI expressed in Figure 3C indicated the closeness of both values.

#### 3.1.3. Effect of Independent Variables on Entrapment Efficiency

The entrapment efficiency of all the batches was determined in a triplicate manner and the results are summarized in Table 1. The effect of formulation variables was studied on entrapment efficiency. The ANOVA suggested the quadratic model, and F-value of 26.3 (*p* < 0.0500) implied the model was significant. The F-value of 0.31 implied that the lack of fit was not significant relative to the pure error. Non-significant lack of fit was desired; we wanted the model to fit (Table 2). The following coded equation describes the relation between independent factors and entrapment efficiency.

Entrapment efficiency = +94.99 + 0.4625A − 0.30B − 0.21C − 0.93AB + 0.95AC − 2.10BC − 0.85A² + 2.52B² − 1.47C²(6)

**Figure 4:**
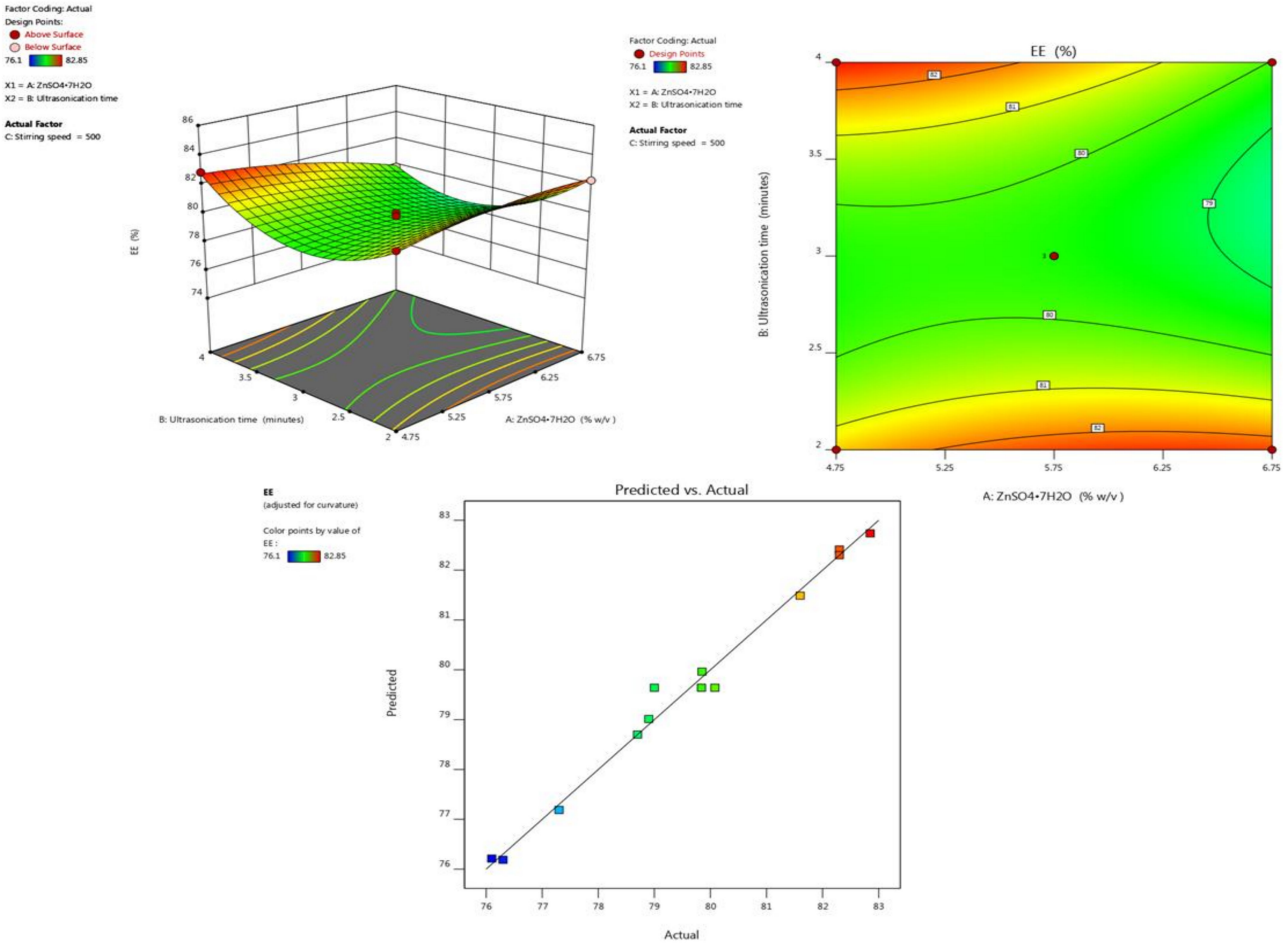
3D (**A**), contour (**B**), and actual and predicted (**C**) values of particle size.

The equation showed in this case that AB, AC, BC, B², and C² are significant model terms. It indicated that Factor A had a positive effect, and B and C had a negative effect on entrapment efficiency. Interaction terms (AB, BC) showed a positive effect, and AC had a negative effect on entrapment efficiency. However, higher-order terms (A^2^ and C^2^) showed a negative effect, and B^2^ had a positive effect on entrapment efficiency. The effect of independent variables on the entrapment efficiency is graphically expressed in the 3-D and contour plot in Figure 4A,B. The plots indicate that, by increasing the concentration of ZnSO_4_·7H_2_O, entrapment efficiency increased, whereas when increasing the ultra-sonication time and stirring speed, the entrapment efficiency decreased because leaching of the drug takes place during the breaking of particles.

#### 3.1.4. Selection of Optimized Formulation

The optimized formulation was chosen from the point prediction of the software, which explained the center point of the formulation. The composition of the optimized formulation had 5.75% *w*/*v* of ZnSO_4_·7H_2_O, at 3.00 min ultrasonic time and 500 rpm stirring speed. The predicted value was 79.731% EE, 259.38 nm particle size, and 0.280 PDI. The experimental value of the optimized formulation was found to be 257.1 ± 1.76 particle size, 79.00 ± 0.33% entrapment, and 0.289 ± 0.003 efficiency. The actual value showed less deviation from the predicted value.

### 3.2. Particle Size, Zeta Potential and PDI

**Figure 5:**
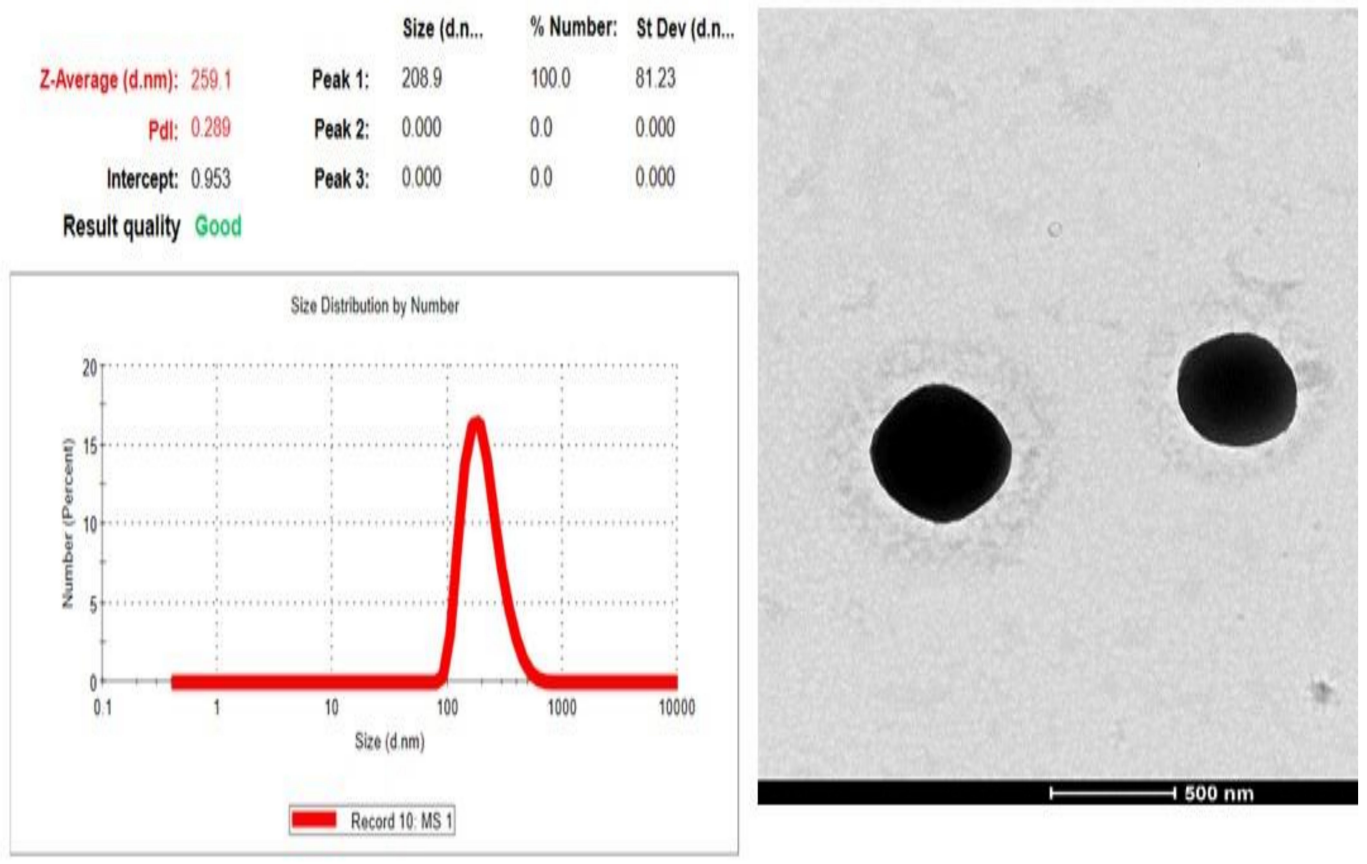
Particle size, zeta potential, and PDI value and morphological examination.

The particle size, zeta potential, and PDI of ZnO-MJE-NPs were found to be 257.1 ± 1.76 nm, −22.7 mV, and 0.289 ± 0.003, respectively (Figure 5). The PDI value of <0.5 indicated homogeneous distribution and uniform particle sizes. The zeta potential of −22.7 mV indicated that the particle was in disaggregated form and stable.

### 3.3. Morphological Examination

The morphology of ZnO-MJE-NPs was analyzed by TEM and showed that the nanoparticles were spherical in shape, with uniform size and disaggregated form (Figure 5).

### 3.4. In Vitro Release Studies

**Figure 6:**
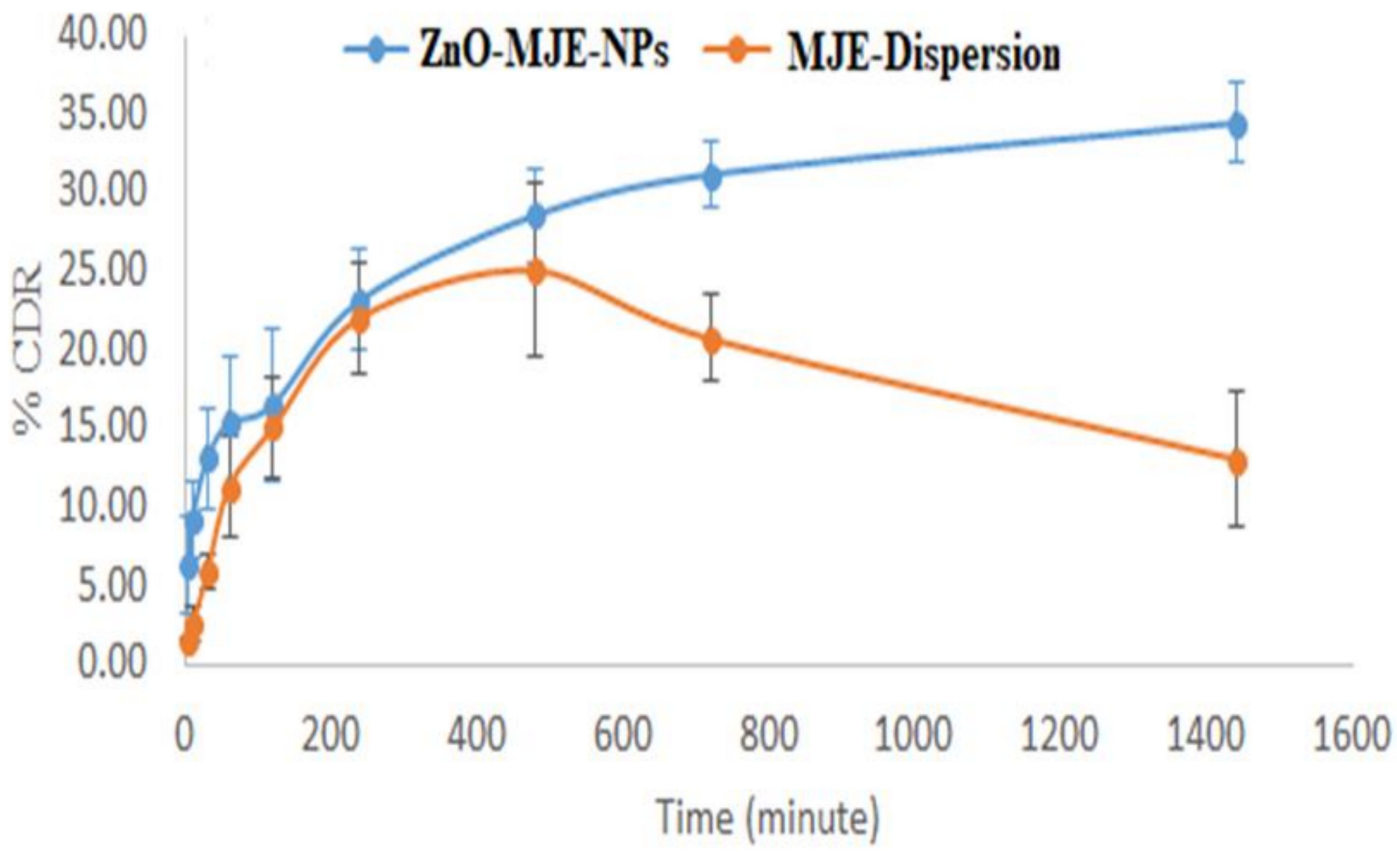
Comparative releases of the drug at pH 7.4.

Figure 6 depicts the release of MJE from ZnO-MJE-NPs-opt at pH 7.4 and compares it with the MJE dispersion. The ZnO-MJE-NPs-opt dispersion and MJE dispersion exhibited 34.5 ± 0.25% and 13.11 ± 2.32% MJE release, respectively, at pH 7.4. Data obtained by the in vitro drug release of ZnO-MJE-NPs-opt were fitted to various drug release kinetic models such as the zero order model, first order model, Higuchi model, and Korsmeyer–Peppas model. The correlation coefficient (R^2^) was calculated and is shown in Table 3. The maximum R^2^ value indicated the Korsmeyer–Peppas model was considered as a best-fit model. The value of “n” was found to be 0.273 (pH 7.4), which indicated that drug release from the optimized formulation followed quasi-Fickian diffusion, i.e., non-swellable matrix diffusion [38].

### 3.5. Ex Vivo Studies

**Figure 7:**
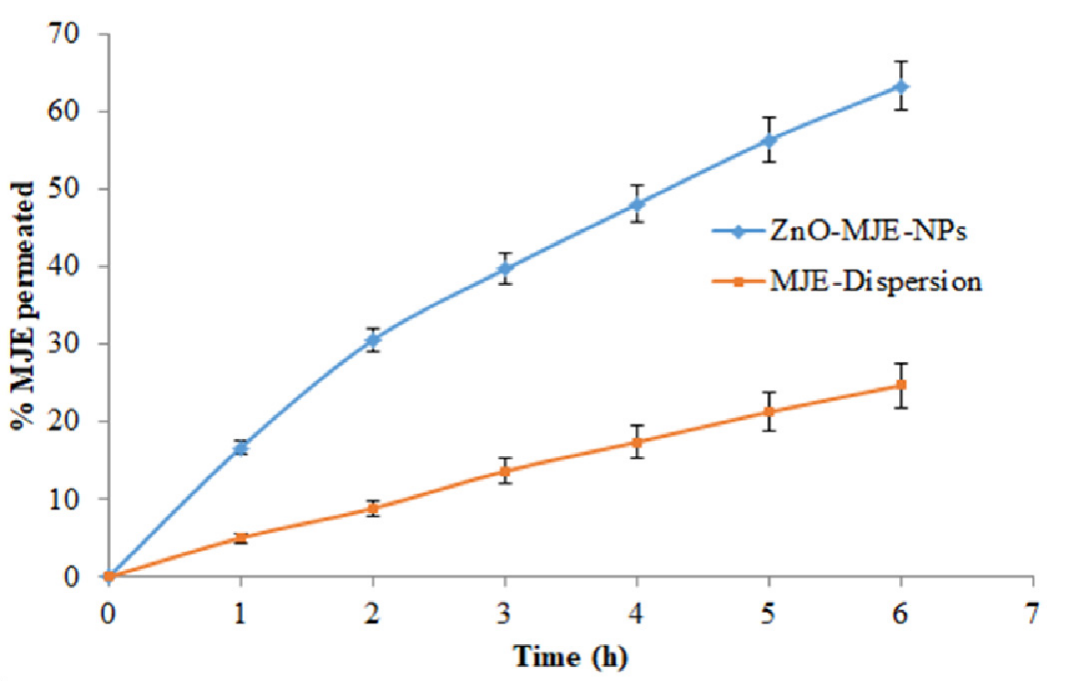
Comparative permeation of ZnO nanosuspension and MJE suspension.

The permeation study of the ZnO-MJE-NPs-opt and MJE dispersion was done on rat skin for 6 h and data are shown in Figure 7. The percentages of cumulative drug permeated across the rat skin were found to be 24 and 63.26% for the MJE dispersion and ZnO-MJE-NPs-opt, respectively. The PC values of the ZnO-MJE-NPs-opt and MJE dispersion were found to be 1.97 × 10^−1^ and 5.497 × 10^−2^, respectively. The ER of ZnO-MJE-NPs-opt was 3.60-fold higher than the MJE dispersion. Thus, it can be assumed that the MJE nanoparticles helped to increase the permeation across the skin, and hence, a better therapeutic response can be expected as compared to the MJE dispersion [43].

### 3.6. Antimicrobial Study

The antimicrobial activity of ZnO-MJE-NPs and MJE was evaluated on *S. Aureus* and *E. Coli.* The ZOI values of ZnO-MJE-NPs were found to be 14 ± 2 and 18 ± 1 mm against *S. Aureus* and *E. Coli,* respectively, at 24 h. The pure MJE showed a ZOI of 9 ± 1 mm against *S. Aureus* and 12 ± 2 mm against *E. Coli* at 24 h. The significantly (*p* < 0.05) high efficacy of ZnO-MJE-NPs was due to the higher solubility, nanosize, as well as penetration in cells of the microorganism than pure MJE. The difference in the activity was due to the permeability barrier provided by the presence of the cell wall [44,45]. The blend of MJE and ZnO had a synergistic effect on antibacterial activity.

### 3.7. Antioxidant Activity

**Figure 8:**
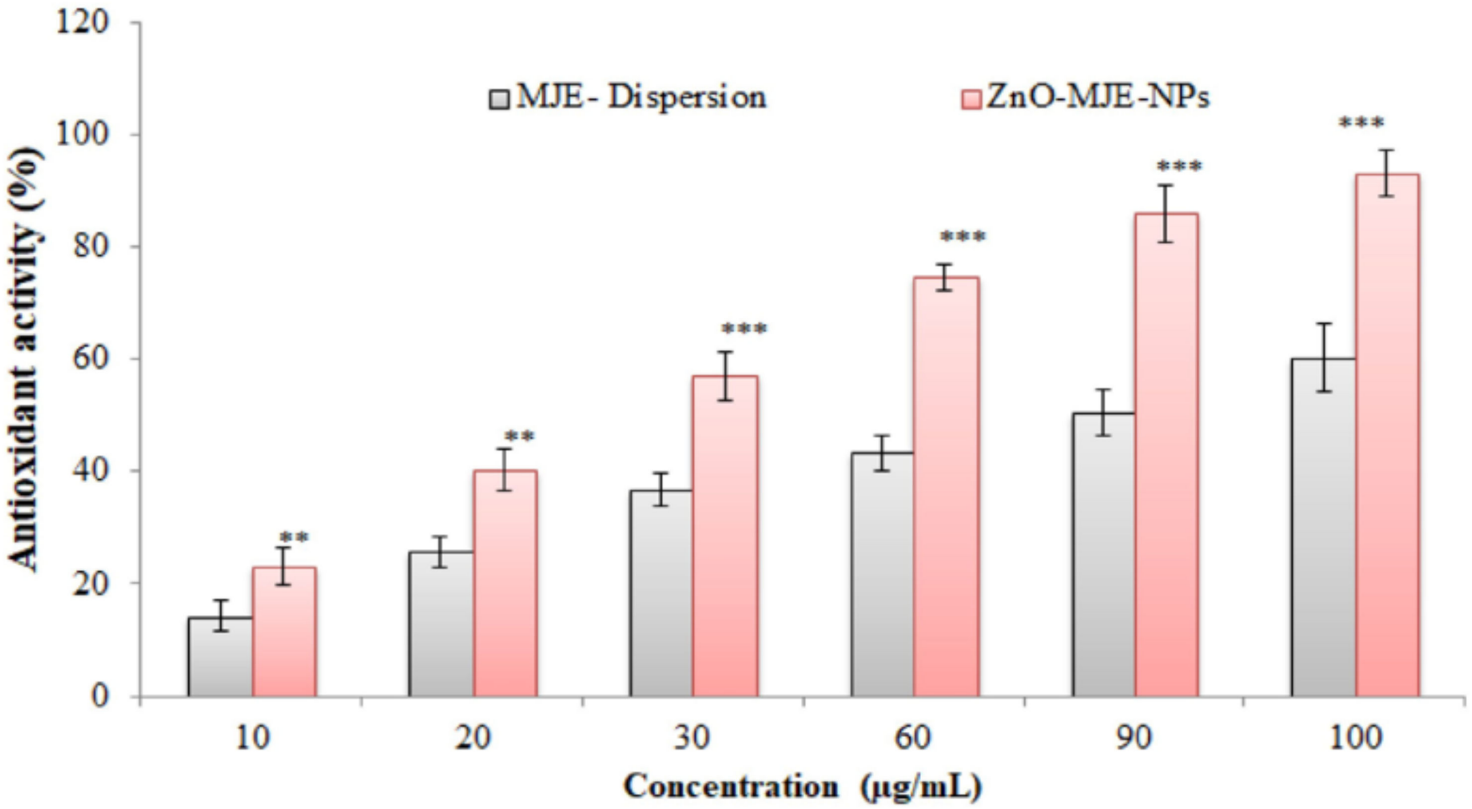
Antioxidant activity of MJE dispersion and ZnO-MJE-NPs. ** *p* < 0.001, *** *p* < 0.0001.

The antioxidant activity of the prepared ZnO-MJE-NPs was evaluated and compared with the MJE extract, and data are shown in Figure 8. It was observed that by increasing the concentration of MJE, the antioxidant activity increased. Significantly (*p* < 0.05) higher antioxidant activity was found in the optimized ZnO-MJE-NPs than in the pure MJE dispersion. The antioxidant activity of ZnO-MJE-NPs was shown to be highest (93.14 ± 4.05%) at 100 µg/mL concentrations; however, the MJE dispersion showed 60.31 ± 6.05% at 100 µg/mL. The significantly (*p* < 0.05) high activity of MJE in ZnO-MJE-NPs was due to the high solubility of MJE. MJE has antioxidant and robust scavengers of free radicals [46]. The antioxidant activity was enhanced by the increased solubility of MJE in ZnO-MJE-NPs.

### 3.8. Stability Studies

Stability of nanoformulation is an important requirement for the shelf life of the formulation. The parameters of the stability study of MJE nanoparticles are given in Table 4, and they were within limits, which indicated that nanoformulation was stable for 3 months.

## 4. Discussion

Green synthesis of ZnO-NPs and Ag-NPs has previously been reported for *Rubia cordifolia*. In this study, the preparation method for the nanoparticles was modified, which helped in achieving a better drug release. A new approach to optimization of the formulation method using BBD lessened the laboratory work with an efficient result. A study carried out for the type of response surface using Box–Behnken design, where a quadratic model was followed, indicated that the result of the optimization of the formulation was good when compared to previous studies. The stability studies of the formulation explained the shelf life of the drug. In vitro and ex vivo comparative drug release studies in the present research indicated that the therapeutic effects of transdermal delivery are good. The nanosize of the particles enhanced their permeability and solubility, which increased the therapeutic effects. This was confirmed by the antimicrobial and anti-oxidant activity.

The overall result of this research will be helpful for further research based on different dosage forms as well as pharmacological activities.

## 5. Conclusions

It was concluded that the Design-Expert software assisted in the development and optimization of the ZnO nanoparticles MJE formulation. The in vitro release results showed that the ZnO nanoparticle formulation has a better release profile. The optimized formulation’s skin permeation and stability study results indicated that this nanoformulation has better therapeutic effects. The ZnO nanoparticles showed better in vitro release, antimicrobial and antioxidant activities, and this optimized formulation may have high therapeutic value.

## Figures and Tables

**Table 1:** Observed responses of different formulation batches on the basis of independent variables.

	Factor			Response		
Formulation Code (FC)	ZnSO_4_·7H_2_O (% *w*/*v*)	Ultrasonic Time (min)	Stirring Speed (rpm)	% Entrapment Efficiency ± SD (n = 3)	Particle Size ± SD (n = 3) nm	PDI ± SD (n = 3)
F1	4.75	2	500	81.6 ± 0.17	264.1 ± 0.81	0.291 ± 0.001
F2	6.75	2	500	82.3 ± 0.05	263.1 ± 1.55	0.292 ± 0.002
F3	4.75	4	500	82.85 ± 0.13	261.3 ± 1.62	0.294 ± 0.002
F4	6.75	4	500	79.85 ± 0.11	262.3 ± 1.58	0.281 ± 0.003
F5	4.75	3	400	78.9 ± 0.21	263.4 ± 0.84	0.279 ± 0.001
F6	6.75	3	400	76.3 ± 0.29	257.9 ± 0.85	0.288 ± 0.004
F7	4.75	3	600	76.1 ± 0.29	260.2 ± 1.13	0.289 ± 0.003
F8	6.75	3	600	77.3 ± 0.24	264.5 ± 0.57	0.279 ± 0.001
F9	5.75	2	400	78.7 ± 0.33	257.5 ± 0.57	0.319 ± 0.002
F10	5.75	4	400	82.3 ± 0.24	260.3 ± 0.82	0.315 ± 0.002
F11	5.75	3	500	80.08 ± 0.34	259.1 ± 1.16	0.289 ± 0.001
F12	5.75	3	500	79.84 ± 0.28	260.1 ± 0.65	0.289 ± 0.002
F13 *	5.75	3	500	79.00 ± 0.33	257.1 ± 1.76	0.289 ± 0.003

(* Optimized formulation, SD = standard deviation, n = no. of times).

**Table 2:** ANOVA of responses (particle size, PDI, and entrapment efficiency).

	Particle Size					
Source	Sum of Square	DF	Mean Square	F-Value	*p*-Value	
Model	64.30	9	7.14	23.30	0.0126	Significant
A-ZnSO_4_·7H_2_O	0.1800	1	0.1800	0.5870	0.4994	--
B-Ultra-Sonication Time	3.24	1	3.24	10.57	0.0475	--
C-Stirring Speed	2.89	1	2.89	9.42	0.0546	--
AB	1.00	1	1.00	3.26	0.1687	--
AC	24.01	1	24.01	78.29	0.0030	--
BC	7.05	1	7.05	23.00	0.0172	--
A²	16.11	1	16.11	52.52	0.0054	--
B²	1.52	1	1.52	4.95	0.1126	--
C²	0.4563	1	0.4563	1.49	0.3097	--
Residual	0.9200	3	0.3067	--		--
Lack of Fit	0.1800	1	0.1800	0.4865	0.5577	Not Significant
Pure Error	0.7400	2	0.3216	--	--	--
Cor Total	65.22	12	--	--	--	---
		**PDI**				
Model	0.0018	9	0.0002	10.50	0.0393	Significant
A-ZnSO_4_·7H_2_O	0.0000	1	0.0000	1.10	0.3719	--
B-Ultra-sonication time	0.0000	1	0.0000	0.8306	0.4293	--
C-Stirring speed	2.500 × 10^7^	1	2.500 × 10^7^	0.0130	0.9165	--
AB	0.0000	1	0.0000	2.54	0.2090	--
AC	0.0001	1	0.0001	4.68	0.1191	--
BC	2.168 × 10^19^	1	2.168 × 10^19^	1.126 × 10^14^	1.0000	--
A²	0.0006	1	0.0006	30.17	0.0119	--
B²	0.0008	1	0.0008	44.08	0.0070	--
C²	0.0004	1	0.0004	20.77	0.0198	--
Residual	0.0001	3	0.0000	--	--	--
Lack of Fit	0.0000	1	0.0000	0.7090	0.4884	Not significant
	0.0000	2	--	--	--	--
	0.0019	12	--	--	--	--
		**Entrapment efficiency**				
Model	58.83	9	6.54	26.34	0.0106	Significant
A-ZnSO_4_·7H_2_O	1.71	1	1.71	6.90	0.0786	--
B-Ultra-sonication time	0.3600	1	0.3600	1.45	0.3148	--
C-Stirring speed	0.8100	1	0.8100	3.26	0.1685	--
AB	3.42	1	3.42	13.79	0.0340	--
AC	3.61	1	3.61	14.55	0.0317	--
BC	5.88	1	5.88	23.70	0.0166	--
A²	0.5003	1	0.5003	2.02	0.2507	--
B²	15.23	1	15.23	61.36	0.0043	--
C²	10.57	1	10.57	42.58	0.0073	--
Residual	0.7445	3	0.2482	--	--	--
Lack of Fit	0.1013	1	0.1013	0.3148	0.6312	not significant
Pure Error	0.6432	2	--	--	--	--
Cor Total	59.58	12	--	--	--	--

**Table 3:** Various kinetic release models.

Dissolution Media	Zero Order		First Order		Higuchi Model		Korsmeyer–Peppas	
-	R^2^	K	R^2^	K	R^2^	K	R^2^	K
pH 7.4	0.8725	0.033	0.9098	0.000	0.9679	1.143	0.9912	4.982

**Table 4:** Result of the stability study of ZnO-MJE-NPs at 25 ± 1 °C/60% RH for 3 months.

Months	Evaluation Parameter			
	Physical Appearance	Particle Size (Mean ± SD) (N = 3)	Entrapment Efficiency (Mean ± SD) (N = 3)	Drug Content (Mean ± SD) (N = 3)
0	Clear and No Sedimentation or Cake Formation.	262.5 ± 2.36	78.78 ± 2.51	97.26 ± 2.17
1	Clear and No Sedimentation or Cake Formation.	265.1 ± 4.17	77.89 ± 1.98	98.56 ± 1.88
2	Clear and No Sedimentation or Cake Formation.	263.7 ± 3.11	76.82 ± 2.28	97.76 ± 2.51
3	Clear and No Sedimentation or Cake Formation.	267.5 ± 2.78	76.23 ± 2.76	97.35 ± 2.51

## Data Availability

The data presented in this work are available in the article.

## Sample Availability

Samples are not available from the author.
